# PTEN in kidney diseases: a potential therapeutic target in preventing AKI-to-CKD transition

**DOI:** 10.3389/fmed.2024.1428995

**Published:** 2024-08-06

**Authors:** Fangfang Cao, Yuanyuan Li, Ting Peng, Yuanmei Li, Lihua Yang, Lanping Hu, Han Zhang, Jiali Wang

**Affiliations:** ^1^Division of Nephrology, Mianyang Central Hospital, Mianyang, China; ^2^Division of Science and Education, Mianyang Central Hospital, Mianyang, China; ^3^Hemodialysis Center, Mianyang Central Hospital, Mianyang, Sichuan, China; ^4^NHC Key Laboratory of Nuclear Technology Medical Transformation (Mianyang Central Hospital), Mianyang, China

**Keywords:** CKD, PTEN, AKI, AKI-to-CKD, kidney

## Abstract

Renal fibrosis, a critical factor in the development of chronic kidney disease (CKD), is predominantly initiated by acute kidney injury (AKI) and subsequent maladaptive repair resulting from pharmacological or pathological stimuli. Phosphatase and tensin homolog (PTEN), also known as phosphatase and tensin-associated phosphatase, plays a pivotal role in regulating the physiological behavior of renal tubular epithelial cells, glomeruli, and renal interstitial cells, thereby preserving the homeostasis of renal structure and function. It significantly impacts cell proliferation, apoptosis, fibrosis, and mitochondrial energy metabolism during AKI-to-CKD transition. Despite gradual elucidation of PTEN’s involvement in various kidney injuries, its specific role in AKI and maladaptive repair after injury remains unclear. This review endeavors to delineate the multifaceted role of PTEN in renal pathology during AKI and CKD progression along with its underlying mechanisms, emphasizing its influence on oxidative stress, autophagy, non-coding RNA-mediated recruitment and activation of immune cells as well as renal fibrosis. Furthermore, we summarize prospective therapeutic targeting strategies for AKI and CKD-treatment related diseases through modulation of PTEN.

## 1 Introduction

Acute kidney injury (AKI) is a clinical syndrome characterized by acute renal failure and poor clinical prognosis, which results in evident acid–base imbalance, disturbances in water and electrolyte homeostasis, and increases nitrogenous waste products in the blood, known as azotemia (1). The development of AKI is frequently precipitated by risk factors such as ischemia–reperfusion injury, cisplatin exposure, and sepsis (2–4). It is distinguished by tubular cell necrosis, tissue damage, renal dysfunction, and the manifestation of acute kidney failure (5). The pathophysiology of renal injury in AKI encompasses a spectrum of mechanisms including autophagy dysregulation, apoptosis induction, oxidative stress response activation, and inflammatory reactions triggered by direct harm to renal vessels and tubular epithelial cells (6). The duration and severity of AKI are highly associated with the progression toward chronic kidney disease (CKD). Inadequate repair post-AKI leads to alterations characterized by tubular atrophy and interstitial fibrosis, ultimately precipitating CKD which involves an array of multiple cell types including fibroblasts, proximal tubular cell, and immune cells (7). Despite extensive research reports on the molecular mechanisms driving the progression from AKI to CKD, the understanding of its pathogenesis and treatment options remains limited, predominantly due to the intricate interplay of various factors involved in this transition. Thus, it is imperative to elucidate their interconnected roles.

The tumor suppressor, phosphatase and tensin homolog (PTEN), located on chromosome 10, is recognized for its dual-specificity phosphatase activity (8). In physiological conditions, PTEN serves as a critical regulator of the PI3K/AKT signaling pathway in inflammatory diseases, influencing critical biological functions such as angiogenesis, cell survival, and other biological processes (9). As a defender against cancer, PTEN inhibits tumorigenesis and tumor progression across various tumors including gastric, liver, ovarian, and kidney cancers, by modulating key cellular functions like cellular proliferation, apoptosis, migration, and invasion (10–12). Importantly, accumulating evidence underscores the significance of PTEN in kidney diseases, particular emphasis on regulating renal energy metabolism, fibrosis, and podocyte injury (13). Adequate expression of PTEN within renal tissue impedes glomerulosclerosis, tubular epithelial cell transdifferentiation, and extracellular matrix accumulation which contribute to renal fibrosis (14). Moreover, beyond its regulatory effects on regulating immune cell recruitment and activation along with autophagy and mitochondrial function during AKI, PTEN also assumes a pivotal position in CKD induced by impaired repair mechanisms through modulating epithelial-mesenchymal transition (EMT) (15, 16). However, the precise mechanisms underlying the contribution of PTEN to AKI pathogenesis as well as AKI-to-CKD transformation remain largely elusive. This review aims to comprehensively summarize the functions and mechanisms associated with PTEN during pharmacological, ischemia–reperfusion, and sepsis-induced AKI as well as AKI-to-CKD transformation. We also discuss potential therapeutic approaches targeting PTEN for treating both AKI and CKD.

## 2 Dual roles of PTEN in AKI

The PTEN consists of nine exons and encodes the protein comprising 403 amino acids with phosphatase activity, which dephosphorylates PI3P to PI2P, resulting in the inactivation of the phosphatidylinositol 3-kinase (PI3K)/Akt signaling pathway (17). The involvement of PTEN-mediated inflammation and apoptosis in the pathogenesis of ischemia–reperfusion injury (IRI)-induced AKI has been widely reported (18). However, the precise mechanism by which PTEN operates in AKI remains elusive, particularly regarding its expression varies under different AKI-provoking situations, both in physiological and pharmacological conditions. Furthermore, the debate continues whether PTEN exerts a protective effect on renal cells or induces renal injury, and controversies exist in the internal molecular mechanisms underlying PTEN’s role in AKI.

### 2.1 PTEN promotes autophagy in alleviating AKI

**Figure 1:**
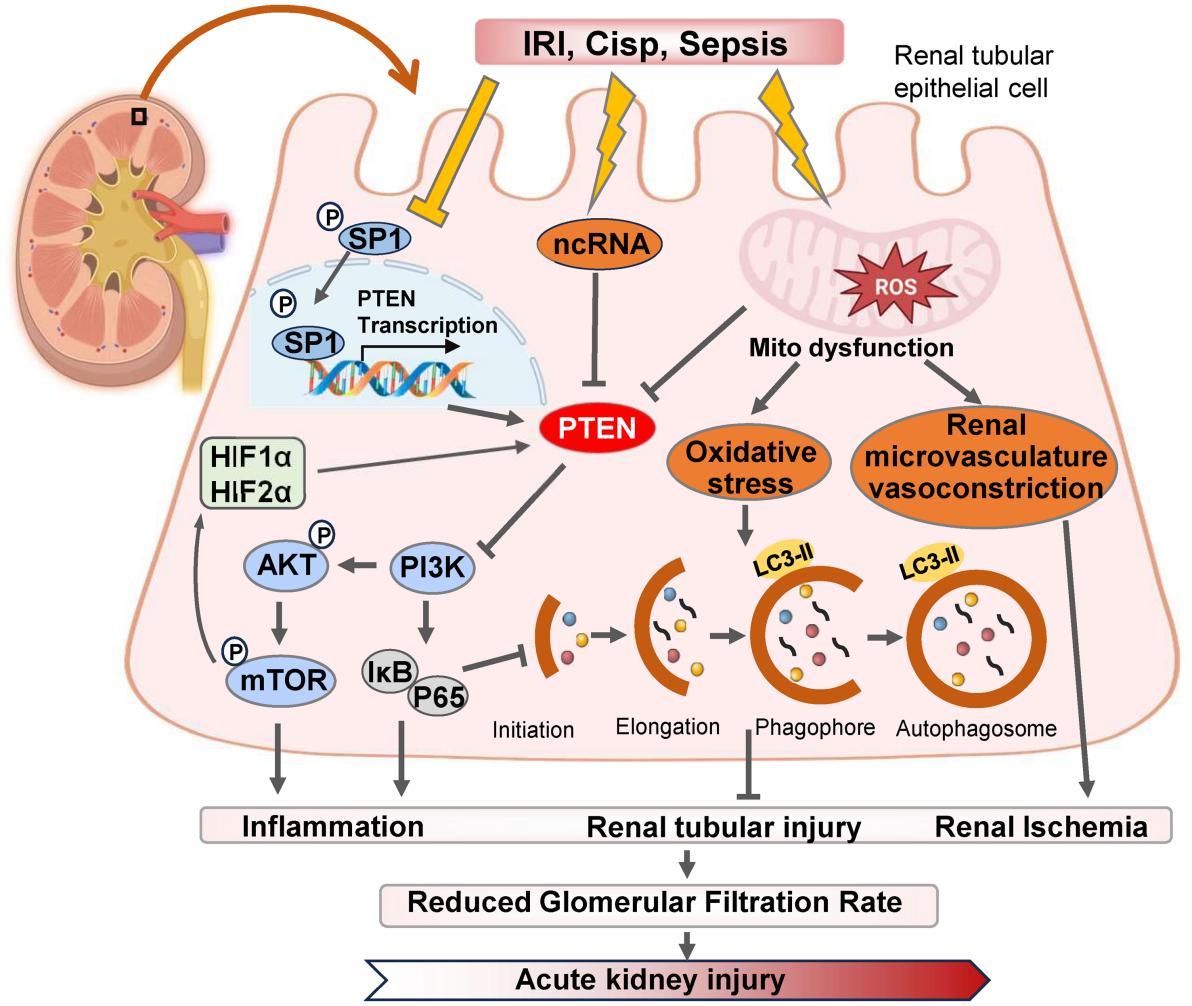
The pathways and molecular mechanisms of PTEN in the progression of AKI. Various pharmacological or pathological factors including ischemia–reperfusion, cisplatin-induced nephrotoxicity, and sepsis, can induce alterations in the expression of SP1 transcription factor, non-coding RNA (ncRNA) molecules, and reactive oxygen species (ROS) within renal tubular epithelial cells. These changes subsequently modulate PTEN expression either positively or negatively. Consequently, the PI3K/AKT/mTOR cascade reaction is triggered and leads to downstream gene modifications associated with inflammation, autophagy, and oxidative stress. Ultimately, these detrimental effects contribute to renal tubular injury and exacerbates AKI. IRI, ischemia–reperfusion injury; Cisp, Cisplatin.

In AKI, renal inflammation is frequently accompanied by the generation of a substantial amount of reactive oxygen species (ROS) and excessive responses to oxidative stress, leading to detrimental effects on mitochondrial structure and function, ultimately exacerbating renal injury (Figure 1) (19). Recent evidence has increasingly demonstrated a close association between autophagy and renal diseases (20, 21). Autophagy represents a dynamic cyclic process wherein damaged or aging macromolecules and organelles within cells are degraded and recycled by proteolytic enzymes in lysosomes to renew cellular components and maintain cellular homeostasis (22). The alterations in autophagy have been extensively documented in AKI induced by cisplatin, ischemia–reperfusion injury (IRI), sepsis, and other risk factors, particularly evident in proximal tubular cells of the kidney (23, 24).

Autophagy plays a dual role in the pathogenesis of AKI. On one hand, autophagy contributes to the repair of renal tubular injury by eliminating reactive oxygen species (ROS) and mitigating mitochondrial dysfunction (25). Moreover, it activates PTEN-induced kinase 1 (PINK1)/MFN2/Parkin-mediated mitophagy in M2 macrophages, and induces autophagy as a protective mechanism for renal tubular cells, which represents a promising approach for improving AKI progression (26). Numerous studies have demonstrated that dynamic changes in autophagy are also crucial for the proliferation and repair of renal tubular cells during the recovery phase following AKI (27). IRI serves as the primary etiology of AKI, leading to necrosis of tubular epithelial cells and triggering an innate immune response (28). Wang et al. (29) established both *in vitro* and *in vivo* models of renal IRI using bilateral renal artery IRI mice and HK-2 cell hypoxia/reoxygenation (H/R), and found that downregulation of PTEN expression in IRI-induced kidney injury, with time-dependent inhibition observed after H/R-induced hypoxia. However, reoxygenation did not affect PTEN expression levels. PTEN exerts renoprotective effects against renal IRI by reducing apoptosis in HK-2 cells while enhancing autophagy concurrently (30). The renoprotective mechanism mediated by PTEN involves activation of PI3K/Akt/HIF1-α and PI3K/Akt/mTOR signaling pathways, which, respectively, mitigate apoptosis and enhance autophagy (Figure 1) (31).

Conversely, excessive activation of autophagy hampers the cellular capacity to eliminate oxygen free radicals, leading to apoptosis under severe stress conditions (32). Research has demonstrated that the PTEN inhibitor BpV1 in combination with phosphoinositide phosphatase myotubularin related protein 14 (MTMR14) can attenuate the interaction between PTEN and LC3B, thereby reducing excessive autophagy and preventing cell death caused by its overactivation, which also exhibits neuroprotective effects in cases of ischemic stroke (33). Furthermore, hyperactivation of autophagy contributes to maladaptive repair in AKI, promoting its progression into CKD (34). Interventions involving PTEN inhibitors protect against oxidative stress-induced damage during sepsis-induced AKI recovery in mice; however, overexpression of PTEN yields contrasting results (35). For renal tubular epithelial cells experiencing severe AKI, maintaining a moderate level of autophagy can mitigate mitochondrial damage and facilitate renal function recovery. Additionally, knockdown of PTEN improves tissue dysfunction by enhancing mitochondrial oxidative phosphorylation, particularly through modulation of autophagic flux (36, 37). Therefore, inhibiting the upregulation of PTEN protein mediated by excessive activation of autophagy represents a potential therapeutic approach for alleviating kidney injury-related diseases especially AKI.

### 2.2 Oxidative stress and inflammation in PTEN-mediated improvement of AKI

The pathogenesis of sepsis-induced AKI involves the interplay of inflammation, oxidative stress, multiple effector cells, and apoptosis as pivotal contributors (38). In septic patients, inflammation not only triggers apoptosis, necrosis, and immune cell recruitment but also exacerbates renal tubular injury by augmenting oxidative stress levels through the generation of reactive oxygen species during the injurious process (39). Therefore, therapeutic interventions targeting oxidative stress, inflammation, and apoptosis hold substantial potential for enhancing outcomes in AKI patients.

Conversely, Zhou et al. presented a contrasting perspective by demonstrating that the upregulation of PTEN expression plays a crucial role in the regulation and protection against AKI induced by ischemia–reperfusion injury (40). In models of AKI induced by ischemia–reperfusion, an increase in PTEN expression was observed, and inhibiting PTEN further exacerbated renal dysfunction following ischemia–reperfusion injury (41). Moreover, Fang et al. (42) also found that PTEN facilitated tubular epithelial cell apoptosis and enhanced Caspase 3 activation in the kidney post-ischemia–reperfusion injury. Conversely, inhibition of PTEN resulted in heightened neutrophil and macrophage infiltration into the kidney, elevated expression of renal inflammatory chemokines and cytokines, ultimately leading to kidney injury (42, 43). Similarly, during renal ischemia–reperfusion and hypoxia in cultured mouse renal cells, miR-687 induced by HIF-1 upregulated PTEN activity to activate the cell cycle and promote apoptosis, which facilitated renal tubular cell proliferation and kidney repair (44). Retaining PTEN attenuated these effects thereby providing protection against kidney injury (9). In summary, PTEN regulates both renal tubular cell apoptosis and inflammation indicating its potential as a therapeutic target for ischemic AKI, modulation of autophagy through gene expression alterations may ameliorate cellular damage in AKI.

### 2.3 Non-coding RNA (ncRNA)-targeted PTEN in AKI

Aberrant expression of long non-coding RNAs (lncRNAs) has been documented in the peripheral blood of AKI patients, indicating their potential as prognostic indicators for AKI patient survival (45). Specifically, the endogenous antisense lncRNA level in the circulating system can serve as one such indicator. Both lncRNA6406 and PTEN expression exhibited downregulation in an LPS-induced AKI model. Moreover, ectopic overexpression of lncRNA 6,406 significantly upregulated PTEN expression, resulting in attenuation of LPS-induced inflammation, inhibition of oxidative stress and apoptosis, ultimately leading to amelioration of AKI (46). This effect is attributed to the role played by lncRNA 6,406 as a competing endogenous RNA (ceRNA), which sequesters miR-687 and modulates LPS-induced AKI through regulation of the miR-687/PTEN axis (47). Targeting lncRNAs-mediated PTEN may provide a novel therapeutic approach for sepsis-induced AKI with a protective effect. Conversely, elevated levels of PTEN can also contribute to kidney injury in AKI (48). Wang et al. (49) demonstrated that overexpression of miR-22-3p *in vivo* could downregulate PTEN expression levels and inhibit inflammation, identifying PTEN as a potential downstream functional regulator controlled by miR-22-3p during regulation of sepsis-induced kidney injury (50).

Hypoxia-inducible factor (HIF) levels in ischemia or hypoxia are closely associated with the severity of kidney injury, and accumulating evidence suggests that both HIF and HIF2α exert protective effects in renal ischemia–reperfusion (IR) injury (21, 51). The promoter region of miR-21 contains a binding site for HIF-1α, and miR-21 regulates the expression of HIF-1α by targeting the PTEN/Akt signaling pathway, which is involved in the kidney’s response to IRI (52). Song et al. (53) demonstrated that IRI induces high expression of miR-21 in renal tubular epithelial cells, and inhibition of miR-21 exacerbates AKI. Elevated levels of miR-21 suppress PTEN, activate the AKT/mTOR/HIF pathway, and inhibit apoptosis of renal tubular epithelial cells (Figure 1) (53). Furthermore, miR-21 attenuates kidney injury by inhibiting inflammatory responses mediated by mature dendritic cells through the PDCD4/NF-κB pathway (54, 55).

Cisplatin (Cisp) and other platinum derivatives are currently widely used chemotherapeutic agents for the treatment of solid tumors (56). However, dose-dependent toxicity is a significant adverse reaction (57). PTEN exhibits high expression in multiple organ tissues, and studies have reported that inhibiting PTEN can provide protection to intestinal, neuronal, and cardiomyocyte cells (58–60). Inhibition of PTEN can promote PI3K/Akt activation and exacerbate cisplatin-induced AKI (61). Inhibition of PTEN activity aggravates cisplatin-induced AKI and renal tubular cell apoptosis by further activating caspase 3 and upregulating Bax expression levels, which leads to the blockade of p53 signaling pathway activation, ultimately resulting in inflammatory cell infiltration and production of proinflammatory molecules (41, 62). On the contrary, Huang et al.’s study confirmed reduced PTEN expression in a cisplatin-induced AKI model with involvement of the miR-181a/PTEN axis in curcumin’s protective effect against cisplatin-induced AKI. Curcumin treatment reduces miR-181a expression levels induced by cisplatin while restoring inhibited PTEN expression *in vivo* (63). Considering the pivotal role of PTEN in the pathogenesis of cisplatin-induced AKI, regulating its activity represents a novel therapeutic strategy for this condition.

### 2.4 Sp1/PTEN/AKT axis-mediated renoprotection in AKI

The function of PTEN and its downstream proteins is highly dependent on the transcriptional activity of PTEN itself (64). As a critical upstream regulator of PTEN, Sp1 (specific protein 1) belongs to the transcription factor family, which also includes Sp2, Sp3, and Sp4 (61). Sp1 has been closely associated with significant biological processes such as cell growth, differentiation, apoptosis, and carcinogenesis (65). Sp1 inhibits the activity of PTEN by modulating the transcriptional level (66). Furthermore, Sp1 exacerbates diabetic renal tubular injury by binding to the promoter of phosphoglycerate mutase family member 5 (PGAM5) (67). Numerous studies have revealed that SP1 expression and intracellular mitochondrial division are significantly increased in diabetes or hyperglycemia environments (68). Additionally, SP1 is markedly upregulated in type 2 diabetes (T2DM)-induced pulmonary tuberculosis (PTB) (69). Moreover, through Akt activation pathway-mediated signaling cascade regulation mechanism involving SP1 suppresses PTEN transcription while promoting lung injury in T2DM-PTB mice models (70). However, overexpression of PTEN enhances mouse survival rate while reducing inflammatory infiltration, apoptosis, and fibrosis in lung tissue (71).

The downregulation of SP1 expression and upregulation of PTEN expression were observed in the IRI-induced AKI model (72). SP1 modulates autophagy and ameliorates IRI-induced AKI by regulating the miR-205/PTEN axis and mediating the Akt signaling pathway (72). Overexpression of SP1 inhibits the upregulation of PTEN expression, thereby promoting p-AKT activation to mitigate AKI (73). SP1 suppresses mRNA and protein expression of PTEN through repressing transcription at site C (918/913) in the core promoter region of PTEN, while site B (934/929), due to its proximity to the activator binding site (Egr-1) (947/939), cannot mediate SP1 inhibition of PTEN transcription (74–76). Therefore, targeting specific SP1 binding sites on PTEN for selective activation or inhibition may serve as a potential therapeutic strategy for kidney injury-related diseases, and the SP1/PTEN/Akt axis holds promise as a therapeutic approach for ischemia–reperfusion-induced AKI.

## 3 Regulation of PTEN in CKD

The occurrence and progression of renal tubular injury in AKI could result in either complete or incomplete renal recovery (77). Long-term follow-up data indicate that approximately 25% of AKI patients develop renal fibrosis, leading to the development of CKD (15, 78). CKD is characterized as a global public health issue with an increasing incidence rate, and renal interstitial inflammation and fibrosis are the predominant pathological processes involved in CKD progression, ultimately culminating in end-stage renal disease (ESRD) (79–81). Renal fibrosis is characterized by activated and proliferating fibroblasts, infiltration of inflammatory cells, and atrophy of renal tubular cells (82). Epithelial-mesenchymal transition (EMT) induced by excessive accumulation of extracellular matrix (ECM) proteins, including type I and type III collagen within the renal interstitium represents a critical stage (83, 84). However, effective treatment methods to prevent this process are currently limited. It is crucial to comprehend the pathophysiological mechanisms underlying the occurrence and development of renal interstitial fibrosis while exploring novel strategies for preventing and treating CKD leading to ESRD.

### 3.1 PTEN regulates fibroblast proliferation and differentiation in AKI-to-CKD transition

The primary target of PTEN is phosphatidylinositol 3,4,5-triphosphate (PIP3) (85). Pharmacological inhibition of PTEN not only regulates PI3K/Akt signaling and exacerbates AKI caused by ischemia–reperfusion but also contributes to the pathogenesis of renal inflammation and fibrosis by modulating the infiltration of myeloid fibroblasts and immune cells into the kidney (15, 86). Studies have demonstrated that myeloid-specific deletion of the PTEN gene in mice leads to severe proteinuria, renal dysfunction, and fibrosis following angiotensin-converting enzyme therapy (87). Furthermore, PTEN can suppress renal inflammation and fibrosis in angiotensin II (AngII)-induced hypertensive nephropathy through the CXCL16/phosphatidylinositol-3 kinase γ (PI3Kγ) signaling pathway (88). Additionally, myeloid-specific deficiency of PTEN enhances extracellular matrix protein production while promoting myeloid fibroblast accumulation and myofibroblast formation after angiotensin-converting enzyme inhibitor therapy, which is accompanied by increased macrophage and T cell infiltration in mouse kidneys (61, 89).

**Figure 2:**
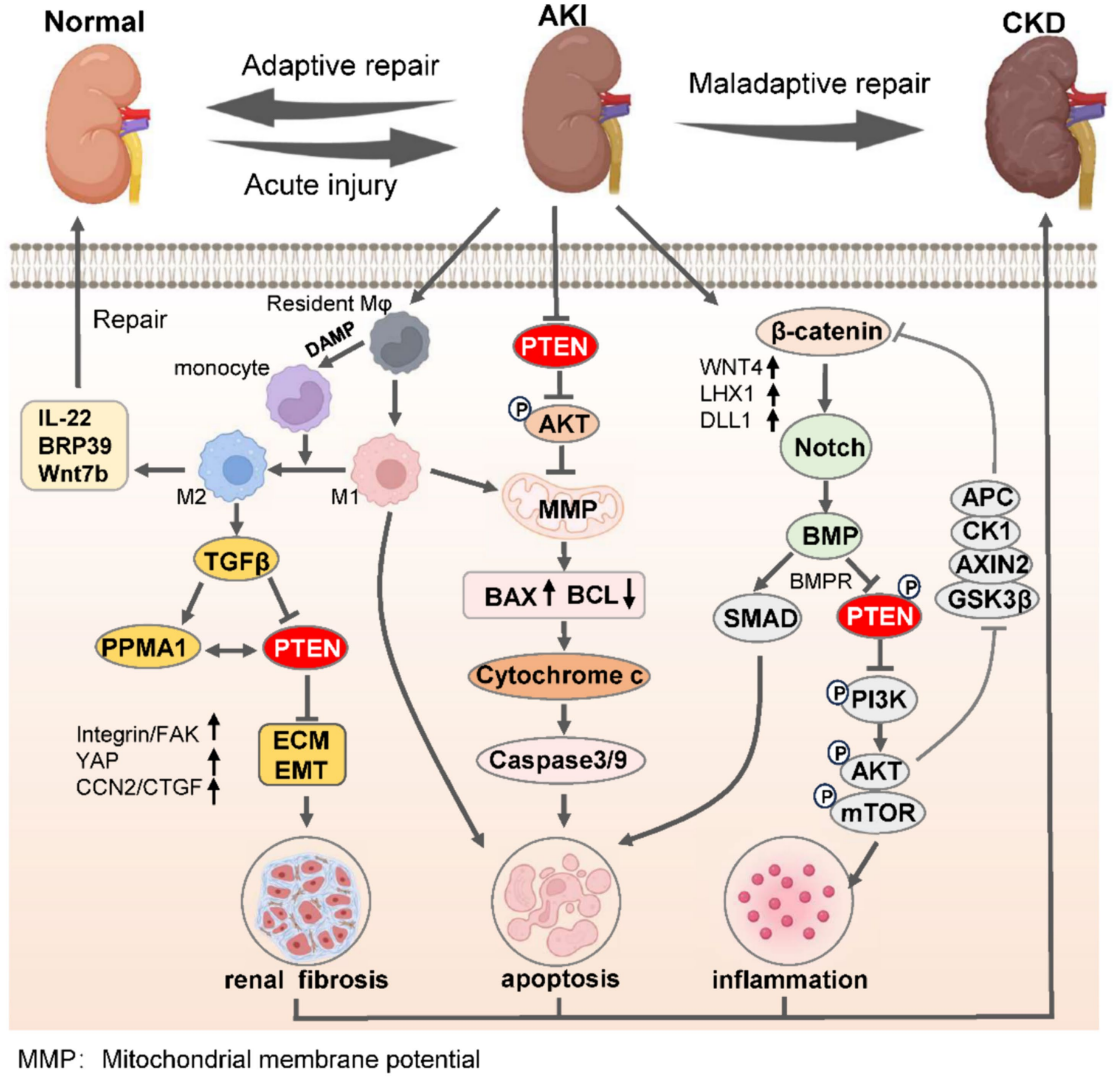
PTEN governs the process of renal adaptive and maladaptive repair in AKI-to-CKD transition. Upon the initiation of AKI, M1 macrophages undergo polarization to M2 phenotypes within the confines of immunoregulatory capacity, leading to the secretion of anti-inflammatory factors to facilitate adaptive kidney repair, thereby mitigating AKI. Conversely, the undue inhibition of PTEN promotes monocyte polarization toward M1 macrophages, suppresses PTEN expression, induces EMT, and enhances renal interstitial fibrosis. Simultaneously, reduced PTEN levels lead to decreased mitochondrial oxidative phosphorylation and mitochondrial membrane permeability, resulting in mitochondrial dysfunction and apoptosis via the caspase protein cascade. Moreover, severe AKI activates the β-catenin/Notch signaling axis to promote BMP-mediated repression of PTEN along with its receptor BMPR, while suppressing inflammatory responses triggered by the PI3K/AKT/mTOR pathway. The synergistic consequences of these effects severely undermine the kidneys’ capacity for effective adaptive restoration, eventually leading to the progression of CKD. ECM, extracellular matrix; EMT, epithelial-mesenchymal transition; AKI, acute kidney injury; CKD, chronic kidney disease.

During the transition from AKI to CKD, the activation of pro-inflammatory cytokines can induce an upregulation of non-coding RNA expression, thereby exacerbating renal injury and promoting fibroblast proliferation and differentiation (73, 90). This process is highly dependent on the activation of the Wnt/β-catenin axis and Notch-induced Bmp signaling, which in turn promotes PTEN-mediated inflammatory cell activation of PI3K/AKT/mTOR (91, 92). Additionally, differences in macrophage polarization directly determine the outcomes of kidney adaptive repair. M2 polarization alleviates kidney fibrosis by inhibiting PTEN expression, while M1 polarization accelerates mitochondrial dysfunction and activates Bcl-2/Bax/Caspase-3 signaling, leading to maladaptive kidney repair (93, 94). The aristolochic acid (AA) nephropathy model serves as a representative model for the progression from AKI to CKD, and targeting the NF-κB/miR-382/PTEN/AKT axis can significantly ameliorate AA-induced tubulointerstitial fibrosis (Figure 2) (95).

### 3.2 PTEN regulates epithelial-mesenchymal transition (EMT)

The renal tubular epithelium is susceptible to various adverse effects including ischemia, toxicology, and pharmacology (96). Under stressful conditions, injured renal tubular epithelial cells can communicate with mesenchymal cells, particularly fibroblasts, and stimulate their activation and proliferation, thereby contributing to the development of renal fibrosis (97). Previous studies have demonstrated that exosome-mediated miR-21 released by renal tubular cells promotes fibroblast activation in ureteral obstruction-induced hydronephrosis and long-term renal fibrosis models, and the activation occurs through targeting PTEN and thus modulating the PTEN/Akt axis (98). Furthermore, enhancer of zeste homolog 2 (EZH2) enhances EMT by binding to the PTEN promoter and regulating its transcription (99). EZH2 also activates the EGFR/ERK1/2/STAT3 signaling pathway and induces M2 macrophage polarization through STAT6 and PI3K/AKT pathways, which exacerbates renal fibrosis (Figure 2) (15, 100).

Ubiquitination of PTEN is crucial in EMT-mediated renal fibrosis as well. During the progression of CKD, EMT promotes the deposition of interstitial matrix (101). In diabetic kidney disease (DKD) mice with renal tubular injury, there is a significant increase in the level of PtenK27-polyUb (13). When PTEN undergoes polyubiquitination at lysine 80 through MEX3C-mediated K27 linkage, it exhibits a pro-inflammatory effect by promoting the secretion of interleukin-6 (IL-6) and transforming growth factor-beta 1 (TGF-β), thereby exacerbating hyperglycemia-induced EMT (102). The MEX3C-PTENK27-polyUb axis also enhances the dephosphorylation and protein stabilization of fibrosis-related proteins TWIST, SNAI1, and YAP in renal tubular epithelial cells, thus aggravating EMT (103). The concentration of PTENK27-polyUb in urine may serve as a potential prognostic indicator for diabetic disease or patients with renal fibrosis complicated by AKI who exhibit a poor prognosis.

### 3.3 PTEN affects BMP and its receptors to regulate fibrosis

Vascular calcification presents a major contributor to cardiovascular disease in patients with CKD (104). Elevated serum levels of bone morphogenetic proteins (BMPs), such as BMP-2 and BMP-4, have been identified as potential serum markers for vascular calcification in CKD (105). Moreover, BMP demonstrates renoprotective effects in various acute and chronic kidney injuries, including IRI, unilateral ureteral obstruction (UUO), and streptozotocin (STZ)-induced injury (106). BMP is predominantly expressed in proximal renal tubular cells and glomerular podocytes (107). Importantly, it plays a significant role in fibrosis during the progression from AKI to CKD (108). As an inhibitor of PI3K and BMP-7, BMP-7 suppresses PI3K/Akt signaling by upregulating PTEN expression without altering fibroblast activation or α-SMA expression (109). This counteracts the profibrotic effect of TGFβ1 on cultured renal cells and prevents interstitial fibrosis in both acute and chronic kidney injury (110).

Endothelial dysfunction is commonly recognized as a characteristic step in vascular injury among patients with CKD (111). Recent findings have indicated that PTEN-mediated activation of BMP receptor signaling serves as a potential mechanism contributing to endothelial dysfunction in CKD (112). The activation of BMP receptors plays a conclusive role in the mechanism underlying endothelial dysfunction during early stages of CKD. PTEN, as a key mediator between BMP receptors and endothelial function, assumes an important role in the development of endothelial dysfunction in CKD (91). As a downstream effector molecule activated by BMP receptor signaling, PTEN is upregulated in vascular endothelial cells of mice with CKD, leading to the activation of BMP receptor signaling and inhibition of the Akt-eNOS pathway, subsequently resulting in endothelial dysfunction (113).

### 3.4 Epigenetic modification mediates PTEN to regulate renal fibrosis

Epigenetic modifications are critically implicated in renal pathologies, exerting substantial influence on the progression of renal fibrosis (114). During ischemia–reperfusion injury, UUO, nephrotoxic drug-AKI, CKD, and the transition from AKI to CKD, epigenetic regulatory mechanisms are activated to varying degrees (103, 115). Histone modification (acetylation and methylation) and DNA methylation are currently the most extensively investigated epigenetic modifications in kidney disease (116, 117). Several histone methyltransferases (HMTs) have been employed to ameliorate renal fibrosis in CKD (118). Studies have demonstrated that activation of epigenetic regulatory factors during diabetic kidney disease progression confers renal protection by restraining inflammation, excessive activation of tubulointerstitial fibroblasts, and fibrosis development (119, 120). In mice with UUO or 5/6 nephrectomy-induced renal fibrosis, there is a notable increase in JMJD3 expression, concomitant with enhanced activities of H3K9 methyltransferase G9a and H3K4 methyltransferase SET7/9 (121). JMJD3 exerts its epigenetic regulation by demethylating H3K27me3 which targets PTEN transcription activation for inhibition (122). The demethylation of H3K27me3 mediated by JMJD3 inhibits AKT phosphorylation through increased PTEN expression, thereby improving renal fibrosis (123). Currently, chemical inhibitors targeting methyltransferases have been reported to significantly reduce the level of H3K9me1 methylation leading to decreased PTEN activity and accelerated renal fibrosis in UUO mice (124).

DNA methylation is a critical epigenetic modification pathway in the progression of kidney-related diseases (125). The methylation of PTEN is highly correlated with the occurrence of CKD, and modulating PTEN activity through DNA methylation also constitutes a fundamental element in genetic modification pathways that affect the progression of CKD (126). PTEN regulates ECM metabolism and impacts the onset and development of CKD-induced fibrosis (Figure 2) (14). PTEN methylation leads to decreased PTEN activity in a mouse model of renal fibrosis induced by UUO (127). Hu et al. (126) has demonstrated that DNA methyltransferases (DNMTs), including DNMT1, DNMT3a, and DNMT3b, are primarily involved in this process. Inhibitors targeting DNMTs can effectively ameliorate abnormal DNA methylation in UUO-induced renal fibrosis (128). DNMT negatively regulates PTEN methylation to modulate the PI3K/AKT signaling pathway (129). Previous studies also demonstrated that pharmacologically reducing the interaction between DNMT3a and PTEN can significantly delay renal fibrosis (128). Additionally, knockdown of DNMT3a restores TGF-β1’s inhibitory effect on PTEN activity in the TGF-β-induced renal fibrosis model mainly through controlling Klotho promoter methylation mediated by DNMT3a (130, 131). Consequently, regulating PTEN activity through epigenetic modification pathways including histone or DNA methylation represents a potential therapeutic approach for improving CKD and kidney diseases.

### 3.5 PTEN controls phagophore closure and regulates autophagy

Autophagy plays a dual role in the repair of injury from AKI to CKD (Figure 1) (132). Sustained activation of autophagy may lead to tubular atrophy and shedding, thereby promoting renal fibrosis (133). On the contrary, autophagy can prevent renal interstitial fibrosis by degrading excessive collagen within cells (134). Recently, it has been reported that PTEN-mediated adaptive renal repair in proximal renal tubular cells (RPTC) is crucial for delaying the transition from AKI to CKD, and its expression is inversely correlated with markers of renal injury and fibrosis (135, 136). PTEN alleviates renal fibrosis by upregulating phagophore closure mediated by the autophagy-related protein CHMP2A (35). CHMP2A belongs to the SNF7 family which regulates protein transport from endosomes to lysosomes (137). During the initiation of autophagy, CHMP2A translocates to the phagophore and facilitates phagocytic closure, thereby inducing lysosomal recruitment and fusion while separating the inner and outer autophagosomal membranes to form double-membrane autolysosomes or autophagolysosomes (138, 139). Downregulation of PTEN in RPTC inhibits CHMP2A expression, leading to endosome sorting complex required for transport (ESCRT)-mediated membrane shedding in a model of DKD resulting from impaired renal repair after IRI (126). Conversely, PTEN downregulation leads to heightened interactions between LC3-II and P62 proteins and immature autolysosomal organelles, reflecting a bottleneck in the macroautophagy, which is accompanied by increased apoptosis in renal proximal tubular cells and escalated interstitial fibrosis (140).

Currently, the mechanism by which PTEN regulates CHMP2A and influences cell autophagy remains unclear. Several studies have demonstrated that inhibition of the PI3K/AKT pathway using LY294002 (a specific inhibitor of PI3K) does not affect the expression of CHMP2A, indicating that PTEN-mediated autophagy lysosomal generation through CHMP2A is independent of the PI3K/AKT axis (141, 142). Additionally, pronounced decrease in nuclear PTEN levels compared to cytoplasmic levels in a mouse model of renal fibrosis, which primarily due to phosphorylation of PTEN serine 113 mediated by ATM (ataxia telangiectasia mutated serine/threonine kinase), leading to its translocation into the nucleus (143). Nuclear PTEN further triggers cascade activation of the JUN-SESN2/AMPK axis, thereby enhancing autophagy flux (144, 145). It is also evident that differential concentrations of PTEN between the nucleus and cytoplasm impact transcriptional regulation of CHMP2A (140, 146). However, further investigation is required to elucidate their regulatory mechanism. PTEN-mediated CHMP2A could be a novel therapeutic approach for renal fibrosis resulting from AKI progression to CKD.

## 4 Potential strategies for targeting PTEN to treat AKI and CKD

The selective inhibitor BpV (HOpic) of PTEN has been demonstrated to activate caspase-3 and enhance renal tubular cell apoptosis, exacerbating ischemia–reperfusion injury-induced acute kidney injury (IRI-AKI) (147, 148). Therefore, restoring PTEN expression and function through pharmacological or genetic manipulation pathways represents a promising strategy for mitigating organ damage. Studies have shown that methylation of the PTEN promoter CpG site can target regulatory factors of PTEN to activate and enhance their transcription, thereby inhibiting cancer cell proliferation (40). Similarly, it has been extensively reported that inhibitors targeting the PI3K/PTEN/Akt/mTOR pathway can ameliorate organ injury during cisplatin chemotherapy (149). mTOR inhibitors such as rapamycin and everolimus can effectively protect ovaries from the toxic effects of cisplatin by relying on inactivation of the PI3K/AKT signal mediated by PTEN (150). Currently, drugs (BKM120, AZD-5363 and Olaparib) targeting Class IA PI3K, AKT/p70S6K/PKA or PARP have been used in treating patients with PTEN-defective cancers such as hematologic malignancies, pancreatic and colorectal cancers, which are already tested in phase I to phase III clinical trials (151). However, the use of specific PTEN agonists or small molecule drugs to intervene in the transition of AKI to CKD has not yet been reported in clinical trials. Further exploration is also needed to identify the role of PTEN in kidney diseases.

Recently, genetic intervention to manipulate PTEN has emerged as a promising treatment for AKI and CKD. For instance, the administration of 3-Deazaneplanocin A (DZNep) has been shown to effectively inhibit the specific binding of EZH2 to the proximal promoter of PTEN, thereby reducing the transcription level of PTEN mRNA and improving the pathological damage of CKD (15). It is important to note that recent studies have demonstrated that reversing the transcriptional repression of PTEN in highly invasive tumors such as melanoma through the CRISPR system can effectively inhibit cancer cell proliferation and migration (152). However, it remains unknown whether targeted activation of PTEN mediated by gene editing influences AKI and CKD. Therefore, exogenous PTEN gene modification may offer a potential new therapy to prevent AKI to AKD transition. Additionally, manipulating miR-21 expression and regulating PTEN expression through delivery of antisense oligonucleotides (ASO) or miRNA mimics also holds promise for the treatment in preventing the kidney failure (153).

Renal tubular injury and subsequent regeneration play a pivotal role in determining the recovery from AKI or its progression to CKD (154). Interventions targeting renal tubular regeneration hold significant potential as therapeutic strategies for AKI (155). The upstream transcription factor ZBTB7A regulates the PTEN/AKT pathway downstream of KLF10 protein, thereby augmenting the proliferation and lumen formation capacity of damaged renal tubular cells and promoting renal tubular regeneration in cisplatin-induced AKI (62, 156). Moreover, given PTEN’s regulatory role in apoptosis and autophagy, certain natural plant extracts have been found to enhance PTEN expression and restore impaired autophagy levels (157). These bioactive compounds frequently demonstrate anti-inflammatory, antioxidant, and antifibrotic properties in other disease models (157, 158). Curcumin exerts a protective effect against cisplatin-induced AKI by targeting microRNAs (such as miR-18a and miR-19b), upregulating PTEN expression, improving mitochondrial function, and reducing the release of proinflammatory factors (IL-6, TNF-α, IL-1β) (Table 1) (159–162). However, due to the complex pharmacological effects of natural drugs and the lack of specific PTEN agonists for clinical treatment of AKI, further research is warranted.

**Table 1:** Summary of recent studies investigating the potential strategies for targeting PTEN in AKI and CKD treatment.

Candidate targets	Species (*in vivo*)	Cells (*in vitro*)	Model/samples	Effect on PTEN	Mechanisms	Drugs and status in clinical trials	Reference
3-DZNeP	EZH2fl/flCdh16-Cre+/− Mice;	HK2 cells	IR and folic acid (FA) induced AKI-to-CKD transition	Downregulation of EZH2 to increase PTEN expression	Inhibits EGFR/ERK1/2/STAT3 axis and the polarization of M2 macrophages via STAT6 and PI3K/AKT pathway	Drugs: Tazemetostat (EPZ-6438); Approved	Zhou et al. (15)
miR-382	miR-382−/− mice	renal tubular epithelial cells	Aristolochic acid (AA)-induced AKI-to-CKD transition	Activation of PTEN/AKT signaling	Inflammation response activation and ECM production	Not reported yet	Wang et al. (95)
miR-21	miR-21−/− mice	renal tubular epithelial cells	LPS-induced sepsis	Upregulation of PDCD4/NF-κB and then activation of PTEN/AKT pathways	Increases anti-inflammatory and anti-apoptotic cytokines	Drugs: Lademirsen; RG-012; ADM-21; Clinical trial phase II	Pan et al. (55)
miR-687	Male C57BL/6 mice	The mouse PT cell (BUMPT-306) line; BU.MPT cells	IR-induced AKI; LPS-induced AKI	miR-687 represses PTEN via HIF-1/miR-687/PTEN signaling	Accelerates apoptosis for tissue remodeling	Not reported yet	Bhatt et al. (44) and Liu et al. (46)
bpV(HOpic)	C57BL/6 mice	The mouse lung fibroblasts (NIH-3 T3)	IR-induced apoptosis	Sustained inhibition of PTEN	Activates AKT signaling to alleviate oxidative stress and elevates glycolysis	Not reported yet	Maidarti et al. (147) and Chauhan et al. (148)
ZBTB7A	Male C57BL/6 J mice	Madine-Darby Canine Kidney (MDCK) cells	IR-induced AKI	Activation KLF10 to inhibit PTEN/AKT axis inhibition	Increases renal function and tubular proliferation	Not reported yet	Zhang et al. (62)
Naringenin	Not mentioned	The 786-O cell line; OS-RC-2 cell line	Renal cell carcinoma (RCC)	Inhibition of the PTEN/PI3K/p-AKT axis	Anti-proliferative and apoptosis	Preclinical	Wang et al. (157)
Curcumin	Male C57BL/6 mice	Human embryonic kidney 293 T cell line	Cisplatin-induced AKI	Downregulation of miR-181a to increase PTEN expression	Protection of mitochondrial bioenergetics and redox balance	Preclinical	Huang et al. (63)
IRF4	LysM-Cre IRF4f/f (myeloid IRF4−/−) mice	Kidney Monocytes/macrophages Murine macrophage RAW 264.7 cells	IR-induced AKI	Inhibition of PTEN to activate PI3K/AKT axis	Increases monocyte recruitment against renal fibrosis	Drugs:Frenlosirsen; NMP; KB-9558; Clinical trial phase 1	Sasaki et al. (43)

## 5 Conclusion and future perspectives

The high prevalence of AKI and CKD is often attributed to maladaptive repair processes. Following AKI, the upregulation of proinflammatory cytokines, including IL-1β, TNFα, and TGF-β, is central to the development of renal pathologies such as tubular atrophy, glomerulosclerosis, interstitial fibrosis, renal ischemia, and capillary loss. These clinical manifestations precipitate renal dysfunction, subsequently diagnosed as CKD. PTEN has garnered considerable attention due to its involvement as a lipid phosphatase in various physiological processes including cell proliferation, apoptosis, and fibrosis during the progression of AKI and CKD. The activation of NF-κB and HIF initiates a cascade effect that significantly contributes to the AKI progression. Furthermore, PTEN-mediated interactions among signaling pathways, including PI3K/AKT, TGF-β/Smad, Notch, and NF-κB, has been reported to participate in the EMT process during the transformation from AKI to CKD. In conclusion, serum PTEN concentration emerges as a potential biomarker for assessing the severity of AKI and its progression to CKD. Targeting PTEN to modulate non-coding RNA expression, autophagy, and oxidative stress in renal tubular cells emerges as an effective strategy for alleviating kidney injury. Future research focused on uncovering the functions and mechanisms of PTEN in kidney diseases holds substantial promise for elucidating the pathophysiology of both AKI and CKD, thereby paving the way for novel therapeutic strategies.

## Author contributions

FC: Writing – original draft. YYL: Writing – original draft. TP: Writing – original draft, Software, Data curation. YML: Writing – original draft, Validation, Data curation. LY: Writing – original draft, Formal analysis, Data curation. LH: Writing – original draft, Software, Data curation. HZ: Writing – original draft, Software, Data curation. JW: Writing – review & editing, Funding acquisition.

## References

[1] Schwager E Ghosh E Eshelman L Pasupathy KS Barreto EF Kashani K. Accurate and interpretable prediction of ICU-acquired AKI. J Crit Care. (2023) 75:154278. doi: 10.1016/j.jcrc.2023.154278, PMID: 36774817 PMC10121926

[2] Yuan Y Zhu L Li L Liu J Chen Y Cheng J. S-Sulfhydration of SIRT3 by Hydrogen Sulfide Attenuates Mitochondrial Dysfunction in Cisplatin-Induced Acute Kidney Injury. Antioxid Redox Signal. (2019) 31:1302–19. doi: 10.1089/ars.2019.7728, PMID: 31218880

[3] Wang S Chen Y Han S Liu Y Gao J Huang Y. Selenium nanoparticles alleviate ischemia reperfusion injury-induced acute kidney injury by modulating GPx-1/NLRP3/Caspase-1 pathway. Theranostics. (2022) 12:3882–95. doi: 10.7150/thno.70830, PMID: 35664065 PMC9131270

[4] Kuwabara S Goggins E Okusa MD. The Pathophysiology of Sepsis-Associated AKI. Clin J Am Soc Nephrol. (2022) 17:1050–69. doi: 10.2215/CJN.00850122, PMID: 35764395 PMC9269625

[5] Dong J Feng T Thapa-Chhetry B Cho BG Shum T Inwald DP. Machine learning model for early prediction of acute kidney injury (AKI) in pediatric critical care. Crit Care. (2021) 25:288. doi: 10.1186/s13054-021-03724-0, PMID: 34376222 PMC8353807

[6] Peerapornratana S Manrique-Caballero CL Gomez H Kellum JA. Acute kidney injury from sepsis: current concepts, epidemiology, pathophysiology, prevention and treatment. Kidney Int. (2019) 96:1083–99. doi: 10.1016/j.kint.2019.05.026, PMID: 31443997 PMC6920048

[7] Zhang L Chen F Dong J Wang R Bi G Xu D. HDAC3 aberration-incurred GPX4 suppression drives renal ferroptosis and AKI-CKD progression. Redox Biol. (2023) 68:102939. doi: 10.1016/j.redox.2023.102939, PMID: 37890360 PMC10638610

[8] Worby CA Dixon JE. PTEN. Annu Rev Biochem. (2014) 83:641–69. doi: 10.1146/annurev-biochem-082411-113907 24905788

[9] Masson GR Williams RL. Structural Mechanisms of PTEN Regulation. Cold Spring Harb Perspect Med. (2020) 10:152. doi: 10.1101/cshperspect.a036152, PMID: 31636093 PMC7050585

[10] Chen CY Chen J He L Stiles BL. PTEN: Tumor Suppressor and Metabolic Regulator. Front Endocrinol. (2018) 9:338. doi: 10.3389/fendo.2018.00338, PMID: 30038596 PMC6046409

[11] Xue W Chen S Yin H Tammela T Papagiannakopoulos T Joshi NS. CRISPR-mediated direct mutation of cancer genes in the mouse liver. Nature. (2014) 514:380–4. doi: 10.1038/nature13589, PMID: 25119044 PMC4199937

[12] Gao Y Chen J Ji R Ding J Zhang Y Yang J. USP25 Regulates the Proliferation and Apoptosis of Ovarian Granulosa Cells in Polycystic Ovary Syndrome by Modulating the PI3K/AKT Pathway via Deubiquitinating PTEN. Front Cell Dev Biol. (2021) 9:779718. doi: 10.3389/fcell.2021.779718, PMID: 34805185 PMC8599287

[13] Li Y Hu Q Li C Liang K Xiang Y Hsiao H. PTEN-induced partial epithelial-mesenchymal transition drives diabetic kidney disease. J Clin Invest. (2019) 129:1129–51. doi: 10.1172/JCI121987, PMID: 30741721 PMC6391108

[14] Xu Z Jia K Wang H Gao F Zhao S Li F. METTL14-regulated PI3K/Akt signaling pathway via PTEN affects HDAC5-mediated epithelial-mesenchymal transition of renal tubular cells in diabetic kidney disease. Cell Death Dis. (2021) 12:32. doi: 10.1038/s41419-020-03312-0, PMID: 33414476 PMC7791055

[15] Zhou X Chen H Hu Y Ma X Li J Shi Y. Enhancer of zeste homolog 2 promotes renal fibrosis after acute kidney injury by inducing epithelial-mesenchymal transition and activation of M2 macrophage polarization. Cell Death Dis. (2023) 14:253. doi: 10.1038/s41419-023-05782-4, PMID: 37029114 PMC10081989

[16] Horrillo AS Villanueva LS Cardenas AC Ramos PM Ortiz A Quiroga B. Infectious consequences of the AKI-to-CKD transition. Clin Kidney J. (2022) 15:2237–44. doi: 10.1093/ckj/sfac178, PMID: 36381366 PMC9664570

[17] Hashemi M Etemad S Rezaei S Ziaolhagh S Rajabi R Rahmanian P. Progress in targeting PTEN/PI3K/Akt axis in glioblastoma therapy: Revisiting molecular interactions. Biomed Pharmacother. (2023) 158:114204. doi: 10.1016/j.biopha.2022.114204, PMID: 36916430

[18] Xie X Yang X Wu J Tang S Yang L Fei X. Exosome from indoleamine 2,3-dioxygenase-overexpressing bone marrow mesenchymal stem cells accelerates repair process of ischemia/reperfusion-induced acute kidney injury by regulating macrophages polarization. Stem Cell Res Ther. (2022) 13:367. doi: 10.1186/s13287-022-03075-9, PMID: 35902956 PMC9331485

[19] Aranda-Rivera AK Cruz-Gregorio A Aparicio-Trejo OE Pedraza-Chaverri J. Mitochondrial Redox Signaling and Oxidative Stress in Kidney Diseases. Biomol Ther. (2021) 11:144. doi: 10.3390/biom11081144, PMID: 34439810 PMC8391472

[20] Cybulsky AV. Endoplasmic reticulum stress, the unfolded protein response and autophagy in kidney diseases. Nat Rev Nephrol. (2017) 13:681–96. doi: 10.1038/nrneph.2017.129 28970584

[21] Lin TA Wu VC Wang CY. Autophagy in Chronic Kidney Diseases. Cells. (2019) 8:61. doi: 10.3390/cells8010061, PMID: 30654583 PMC6357204

[22] Kim KH Lee MS. Autophagy—a key player in cellular and body metabolism. Nat Rev Endocrinol. (2014) 10:322–37. doi: 10.1038/nrendo.2014.35, PMID: 24663220

[23] He FF Wang YM Chen YY Huang W Li ZQ Zhang C. Sepsis-induced AKI: From pathogenesis to therapeutic approaches. Front Pharmacol. (2022) 13:981578. doi: 10.3389/fphar.2022.981578, PMID: 36188562 PMC9522319

[24] Wang P Chen W Zhao S Cheng F. The role of LncRNA-regulated autophagy in AKI. Biofactors. (2023) 49:1010–21. doi: 10.1002/biof.1980, PMID: 37458310

[25] Wang B Wang Y Zhang J Hu C Jiang J Li Y. ROS-induced lipid peroxidation modulates cell death outcome: mechanisms behind apoptosis, autophagy, and ferroptosis. Arch Toxicol. (2023) 97:1439–51. doi: 10.1007/s00204-023-03476-6, PMID: 37127681

[26] Lin C Chen W Han Y Sun Y Zhao X Yue Y. PTEN-induced kinase 1 enhances the reparative effects of bone marrow mesenchymal stromal cells on mice with renal ischaemia/reperfusion-induced acute kidney injury. Hum Cell. (2022) 35:1650–70. doi: 10.1007/s13577-022-00756-8 35962179 PMC9515057

[27] Sun M Li J Mao L Wu J Deng Z He M. p53 Deacetylation Alleviates Sepsis-Induced Acute Kidney Injury by Promoting Autophagy. Front Immunol. (2021) 12:685523. doi: 10.3389/fimmu.2021.685523, PMID: 34335587 PMC8318785

[28] Tang Q Dong C Sun Q. Immune response associated with ischemia and reperfusion injury during organ transplantation. Inflamm Res. (2022) 71:1463–76. doi: 10.1007/s00011-022-01651-6, PMID: 36282292 PMC9653341

[29] Wang Y Wang X Wang H Bao J Jia N Huang H. PTEN protects kidney against acute kidney injury by alleviating apoptosis and promoting autophagy via regulating HIF1-alpha and mTOR through PI3K/Akt pathway. Exp Cell Res. (2021) 406:112729. doi: 10.1016/j.yexcr.2021.112729, PMID: 34242625

[30] Song Y Liu W Zhao Y Zang J Gao H. Fumonisin B1 exposure induces apoptosis of human kidney tubular epithelial cells through regulating PTEN/PI3K/AKT signaling pathway via disrupting lipid raft formation. Toxicon. (2021) 204:31–6. doi: 10.1016/j.toxicon.2021.10.013 34740561

[31] Wang H Jiang Q Kang L Yuan L Chen G Cui X. Rheum officinale and *Salvia miltiorrhiza* inhibit renal fibrosis via miR-21/PTEN/Akt signaling pathway in vitro and in vivo. J Ethnopharmacol. (2023) 304:115928. doi: 10.1016/j.jep.2022.115928, PMID: 36513264

[32] Dong Y Chen H Gao J Liu Y Li J Wang J. Molecular machinery and interplay of apoptosis and autophagy in coronary heart disease. J Mol Cell Cardiol. (2019) 136:27–41. doi: 10.1016/j.yjmcc.2019.09.001 31505198

[33] Pan Q Liu Y Wang G Wen Z Wang Y. MTMR14 protects against cerebral stroke through suppressing PTEN-regulated autophagy. Biochem Biophys Res Commun. (2020) 529:1045–52. doi: 10.1016/j.bbrc.2020.06.096 32819563

[34] Pan P Chen J Xie F Guo Y Liu X Zhang D. Enhancing Nix-dependent mitophagy relieves AKI by restricting TREM-1-mediated hyperactivation of inflammasome in platelets. FASEB J. (2023) 37:e23239. doi: 10.1096/fj.202202144RRR, PMID: 37843818

[35] Sang Z Dong S Zhang P Wei Y. miR-214 ameliorates sepsis-induced acute kidney injury via PTEN/AKT/mTOR-regulated autophagy. Mol Med Rep. (2021) 24:322. doi: 10.3892/mmr.2021.12322, PMID: 34328194 PMC8365606

[36] Zhang C Lin T Nie G Hu R Pi S Wei Z. Cadmium and molybdenum co-induce pyroptosis via ROS/PTEN/PI3K/AKT axis in duck renal tubular epithelial cells. Environ Pollut. (2021) 272:116403. doi: 10.1016/j.envpol.2020.116403, PMID: 33433347

[37] Qu NY Zhang ZH Zhang XX Xie WW Niu XQ. Microvesicles containing microRNA-216a secreted by tubular epithelial cells participate in renal interstitial fibrosis through activating PTEN/AKT pathway. Eur Rev Med Pharmacol Sci. (2019) 23:6629–36. doi: 10.26355/eurrev_201908_18552, PMID: 31378905

[38] Manrique-Caballero CL Del Rio-Pertuz G Gomez H. Sepsis-Associated Acute Kidney Injury. Crit Care Clin. (2021) 37:279–301. doi: 10.1016/j.ccc.2020.11.010, PMID: 33752856 PMC7995616

[39] Molema G Zijlstra JG van Meurs M Kamps J. Renal microvascular endothelial cell responses in sepsis-induced acute kidney injury. Nat Rev Nephrol. (2022) 18:95–112. doi: 10.1038/s41581-021-00489-1, PMID: 34667283

[40] Zhou J Jia L Hu Z Wang Y. Pharmacological Inhibition of PTEN Aggravates Acute Kidney Injury. Sci Rep. (2017) 7:9503. doi: 10.1038/s41598-017-10336-8, PMID: 28842716 PMC5572703

[41] Zhou J Fan Y Tang S Wu H Zhong J Huang Z. Inhibition of PTEN activity aggravates cisplatin-induced acute kidney injury. Oncotarget. (2017) 8:103154–66. doi: 10.18632/oncotarget.20790, PMID: 29262553 PMC5732719

[42] Fang X Zhang J Li Y Song Y Yu Y Cai Z. Malic Enzyme 1 as a Novel Anti-Ferroptotic Regulator in Hepatic Ischemia/Reperfusion Injury. Adv Sci (Weinh). (2023) 10:e2205436. doi: 10.1002/advs.202205436 36840630 PMC10161122

[43] Sasaki K Terker AS Pan Y Li Z Cao S Wang Y. Deletion of Myeloid Interferon Regulatory Factor 4 (Irf4) in Mouse Model Protects against Kidney Fibrosis after Ischemic Injury by Decreased Macrophage Recruitment and Activation. J Am Soc Nephrol. (2021) 32:1037–52. doi: 10.1681/ASN.2020071010, PMID: 33619052 PMC8259665

[44] Bhatt K Wei Q Pabla N Dong G Mi QS Liang M. MicroRNA-687 Induced by Hypoxia-Inducible Factor-1 Targets Phosphatase and Tensin Homolog in Renal Ischemia-Reperfusion Injury. J Am Soc Nephrol. (2015) 26:1588–96. doi: 10.1681/ASN.2014050463, PMID: 25587068 PMC4483585

[45] Liu Z Wang Y Shu S Cai J Tang C Dong Z. Non-coding RNAs in kidney injury and repair. Am J Physiol Cell Physiol. (2019) 317:C177–88. doi: 10.1152/ajpcell.00048.2019, PMID: 30969781

[46] Liu X Zhu N Zhang B Xu SB. Long Noncoding RNA TCONS_00016406 Attenuates Lipopolysaccharide-Induced Acute Kidney Injury by Regulating the miR-687/PTEN Pathway. Front Physiol. (2020) 11:622. doi: 10.3389/fphys.2020.00622, PMID: 32655407 PMC7325890

[47] Zhou P Chen Z Zou Y Wan X. Roles of Non-Coding RNAs in Acute Kidney Injury. Kidney Blood Press Res. (2016) 41:757–69. doi: 10.1159/000450566 27832640

[48] Zhang P Yi L Qu S Dai J Li X Liu B. The Biomarker TCONS_00016233 Drives Septic AKI by Targeting the miR-22-3p/AIFM1 Signaling Axis. Mol Ther Nucleic Acids. (2020) 19:1027–42. doi: 10.1016/j.omtn.2019.12.037, PMID: 32059335 PMC7016165

[49] Wang X Wang Y Kong M Yang J. MiR-22-3p suppresses sepsis-induced acute kidney injury by targeting PTEN. Biosci Rep. (2020) 40:527. doi: 10.1042/BSR20200527 PMC7268257 32412059

[50] Zhang J Chen Q Dai Z Pan H. miR-22 alleviates sepsis-induced acute kidney injury via targeting the HMGB1/TLR4/NF-kappaB signaling pathway. Int Urol Nephrol. (2023) 55:409–21. doi: 10.1007/s11255-022-03321-2, PMID: 35960478 PMC9859886

[51] Liu H Li Y Xiong J. The Role of Hypoxia-Inducible Factor-1 Alpha in Renal Disease. Molecules. (2022) 27:318. doi: 10.3390/molecules27217318 36364144 PMC9657345

[52] Lu L Xu H Yang P Xue J Chen C Sun Q. Involvement of HIF-1alpha-regulated miR-21, acting via the Akt/NF-kappaB pathway, in malignant transformation of HBE cells induced by cigarette smoke extract. Toxicol Lett. (2018) 289:14–21. doi: 10.1016/j.toxlet.2018.02.027, PMID: 29501572

[53] Song N Zhang T Xu X Lu Z Yu X Fang Y. miR-21 Protects Against Ischemia/Reperfusion-Induced Acute Kidney Injury by Preventing Epithelial Cell Apoptosis and Inhibiting Dendritic Cell Maturation. Front Physiol. (2018) 9:790. doi: 10.3389/fphys.2018.00790, PMID: 30013485 PMC6036242

[54] Li C Sun Y Jiang C Cao H Zeng W Zhang X. Porcine circovirus type 2 infection activates NF-kappaB pathway and cellular inflammatory responses through circPDCD4/miR-21/PDCD4 axis in porcine kidney 15 cell. Virus Res. (2021) 298:198385. doi: 10.1016/j.virusres.2021.198385, PMID: 33713752

[55] Pan T Jia P Chen N Fang Y Liang Y Guo M. Delayed Remote Ischemic Preconditioning Confers Renoprotection against Septic Acute Kidney Injury via Exosomal miR-21. Theranostics. (2019) 9:405–23. doi: 10.7150/thno.29832, PMID: 30809283 PMC6376188

[56] Ghosh S. Cisplatin: The first metal based anticancer drug. Bioorg Chem. (2019) 88:102925. doi: 10.1016/j.bioorg.2019.102925 31003078

[57] Abd Rashid N Abd Halim SAS Teoh SL Budin SB Hussan F Adib Ridzuan NR. The role of natural antioxidants in cisplatin-induced hepatotoxicity. Biomed Pharmacother. (2021) 144:112328. doi: 10.1016/j.biopha.2021.112328, PMID: 34653753

[58] Zhang L Shen J Cheng J Fan X. MicroRNA-21 regulates intestinal epithelial tight junction permeability. Cell Biochem Funct. (2015) 33:235–40. doi: 10.1002/cbf.3109, PMID: 25997617

[59] Liu W Xu L Wang X Zhang D Sun G Wang M. PRDX1 activates autophagy via the PTEN-AKT signaling pathway to protect against cisplatin-induced spiral ganglion neuron damage. Autophagy. (2021) 17:4159–81. doi: 10.1080/15548627.2021.1905466, PMID: 33749526 PMC8726717

[60] Liang T Gao F Chen J. Role of PTEN-less in cardiac injury, hypertrophy and regeneration. Cell Regen. (2021) 10:25. doi: 10.1186/s13619-021-00087-3, PMID: 34337686 PMC8326232

[61] Zhou J Zhong J Lin S Huang Z Chen H Tang S. Inhibition of PTEN Activity Aggravates Post Renal Fibrosis in Mice with Ischemia Reperfusion-Induced Acute Kidney Injury. Cell Physiol Biochem. (2017) 43:1841–54. doi: 10.1159/000484070, PMID: 29049990

[62] Zhang Y Bao S Wang D Lu W Xu S Zhou W. Downregulation of KLF10 contributes to the regeneration of survived renal tubular cells in cisplatin-induced acute kidney injury via ZBTB7A-KLF10-PTEN axis. Cell Death Discov. (2023) 9:82. doi: 10.1038/s41420-023-01381-6, PMID: 36878898 PMC9988960

[63] Huang SJ Huang J Yan YB Qiu J Tan RQ Liu Y. The renoprotective effect of curcumin against cisplatin-induced acute kidney injury in mice: involvement of miR-181a/PTEN axis. Ren Fail. (2020) 42:350–7. doi: 10.1080/0886022X.2020.1751658, PMID: 32338107 PMC7241563

[64] Tamguney T Stokoe D. New insights into PTEN. J Cell Sci. (2007) 120:4071–9. doi: 10.1242/jcs.015230 18032782

[65] Fulciniti M Amin S Nanjappa P Rodig S Prabhala R Li C. Significant biological role of sp1 transactivation in multiple myeloma. Clin Cancer Res. (2011) 17:6500–9. doi: 10.1158/1078-0432.CCR-11-1036, PMID: 21856768 PMC4318245

[66] Wu Z Luo J Huang T Yi R Ding S Xie C. MiR-4310 induced by SP1 targets PTEN to promote glioma progression. Cancer Cell Int. (2020) 20:567. doi: 10.1186/s12935-020-01650-9, PMID: 33327965 PMC7745362

[67] Liu X Xu C Xu L Li X Sun H Xue M. Empagliflozin improves diabetic renal tubular injury by alleviating mitochondrial fission via AMPK/SP1/PGAM5 pathway. Metabolism. (2020) 111:154334. doi: 10.1016/j.metabol.2020.154334, PMID: 32777444

[68] Xie J Gong Q Liu X Liu Z Tian R Cheng Y. Transcription factor SP1 mediates hyperglycemia-induced upregulation of roundabout4 in retinal microvascular endothelial cells. Gene. (2017) 616:31–40. doi: 10.1016/j.gene.2017.03.027, PMID: 28341181

[69] Zhang P Li YN Tu S Cheng XB. SP1-induced lncRNA TUG1 regulates proliferation and apoptosis in islet cells of type 2 diabetes mellitus via the miR-188-3p/FGF5 axis. Eur Rev Med Pharmacol Sci. (2021) 25:1959–66. doi: 10.26355/eurrev_202102_25096, PMID: 33660806

[70] Zhao H Shi L Wang X Yu X Wang D. Sp1 transcription factor represses transcription of phosphatase and tensin homolog to aggravate lung injury in mice with type 2 diabetes mellitus-pulmonary tuberculosis. Bioengineered. (2022) 13:9928–44. doi: 10.1080/21655979.2022.2062196, PMID: 35420971 PMC9162029

[71] Vallabhaneni S Liu J Morel M Wang J DeMayo FJ Long W. Conditional ERK3 overexpression cooperates with PTEN deletion to promote lung adenocarcinoma formation in mice. Mol Oncol. (2022) 16:1184–99. doi: 10.1002/1878-0261.13132, PMID: 34719109 PMC8895443

[72] Huang C Chen Y Lai B Chen YX Xu CY Liu YF. Overexpression of SP1 restores autophagy to alleviate acute renal injury induced by ischemia-reperfusion through the miR-205/PTEN/Akt pathway. J Inflamm. (2021) 18:7. doi: 10.1186/s12950-021-00270-y, PMID: 33546692 PMC7863508

[73] Bhatraju PK Zelnick LR Herting J Katz R Mikacenic C Kosamo S. Identification of Acute Kidney Injury Subphenotypes with Differing Molecular Signatures and Responses to Vasopressin Therapy. Am J Respir Crit Care Med. (2019) 199:863–72. doi: 10.1164/rccm.201807-1346OC, PMID: 30334632 PMC6444649

[74] Kou XX Hao T Meng Z Zhou YH Gan YH. Acetylated Sp1 inhibits PTEN expression through binding to PTEN core promoter and recruitment of HDAC1 and promotes cancer cell migration and invasion. Carcinogenesis. (2013) 34:58–67. doi: 10.1093/carcin/bgs336, PMID: 23104175

[75] Huang RP Fan Y Ni Z Mercola D Adamson ED. Reciprocal modulation between Sp1 and Egr-1. J Cell Biochem. (1997) 66:489–99. doi: 10.1002/(SICI)1097-4644(19970915)66:4<489::AID-JCB8>3.0.CO;2-H, PMID: 9282327

[76] Brown JL Grau DJ DeVido SK Kassis JA. An Sp1/KLF binding site is important for the activity of a Polycomb group response element from the Drosophila engrailed gene. Nucleic Acids Res. (2005) 33:5181–9. doi: 10.1093/nar/gki827, PMID: 16155187 PMC1214548

[77] Chou YH Huang TM Chu TS. Novel insights into acute kidney injury-chronic kidney disease continuum and the role of renin-angiotensin system. J Formos Med Assoc. (2017) 116:652–9. doi: 10.1016/j.jfma.2017.04.026, PMID: 28615146

[78] McMahon KR Harel-Sterling M Pizzi M Huynh L Hessey E Zappitelli M. Long-term renal follow-up of children treated with cisplatin, carboplatin, or ifosfamide: a pilot study. Pediatr Nephrol. (2018) 33:2311–20. doi: 10.1007/s00467-018-3976-5, PMID: 30218190

[79] Fu Y Tang C Cai J Chen G Zhang D Dong Z. Rodent models of AKI-CKD transition. Am J Physiol Renal Physiol. (2018) 315:F1098–106. doi: 10.1152/ajprenal.00199.2018, PMID: 29949392 PMC6230729

[80] Andre C Bodeau S Kamel S Bennis Y Caillard P. The AKI-to-CKD Transition: The Role of Uremic Toxins. Int J Mol Sci. (2023) 24:152. doi: 10.3390/ijms242216152, PMID: 38003343 PMC10671582

[81] Aparicio-Trejo OE Avila-Rojas SH Tapia E Rojas-Morales P Leon-Contreras JC Martinez-Klimova E. Chronic impairment of mitochondrial bioenergetics and beta-oxidation promotes experimental AKI-to-CKD transition induced by folic acid. Free Radic Biol Med. (2020) 154:18–32. doi: 10.1016/j.freeradbiomed.2020.04.016, PMID: 32360615

[82] Miguel V Tituana J Herrero JI Herrero L Serra D Cuevas P. Renal tubule Cpt1a overexpression protects from kidney fibrosis by restoring mitochondrial homeostasis. J Clin Invest. (2021) 131:695. doi: 10.1172/JCI140695, PMID: 33465052 PMC7919728

[83] Geng XQ Ma A He JZ Wang L Jia YL Shao GY. Ganoderic acid hinders renal fibrosis via suppressing the TGF-beta/Smad and MAPK signaling pathways. Acta Pharmacol Sin. (2020) 41:670–7. doi: 10.1038/s41401-019-0324-7, PMID: 31804606 PMC7468553

[84] Xiong W Xiong Z Song A Lei C Ye C Su H. UCP1 alleviates renal interstitial fibrosis progression through oxidative stress pathway mediated by SIRT3 protein stability. J Transl Med. (2023) 21:521. doi: 10.1186/s12967-023-04376-0, PMID: 37533052 PMC10399010

[85] Shang M Ni L Shan X Cui Y Hu P Ji Z. MTHFD2 reprograms macrophage polarization by inhibiting PTEN. Cell Rep. (2023) 42:112481. doi: 10.1016/j.celrep.2023.112481, PMID: 37149861

[86] Wenta T Schmidt A Zhang Q Devarajan R Singh P Yang X. Disassembly of alpha6beta4-mediated hemidesmosomal adhesions promotes tumorigenesis in PTEN-negative prostate cancer by targeting plectin to focal adhesions. Oncogene. (2022) 41:3804–20. doi: 10.1038/s41388-022-02389-5, PMID: 35773413 PMC9307480

[87] An C Jiao B Du H Tran M Zhou D Wang Y. Myeloid PTEN deficiency aggravates renal inflammation and fibrosis in angiotensin II-induced hypertension. J Cell Physiol. (2022) 237:983–91. doi: 10.1002/jcp.30574, PMID: 34515350 PMC8810675

[88] Lu S Strand KA Mutryn MF Tucker RM Jolly AJ Furgeson SB. PTEN (Phosphatase and Tensin Homolog) Protects Against Ang II (Angiotensin II)-Induced Pathological Vascular Fibrosis and Remodeling-Brief Report. Arterioscler Thromb Vasc Biol. (2020) 40:394–403. doi: 10.1161/ATVBAHA.119.313757, PMID: 31852223 PMC7059862

[89] Qu C Liu X Ye T Wang L Liu S Zhou X. miR-216a exacerbates TGF-beta-induced myofibroblast transdifferentiation via PTEN/AKT signaling. Mol Med Rep. (2019) 19:5345–52. doi: 10.3892/mmr.2019.10200, PMID: 31059054 PMC6522872

[90] Abdelmageed MM Kefaloyianni E Arthanarisami A Komaru Y Atkinson JJ Herrlich A. TNF or EGFR inhibition equally block AKI-to-CKD transition: opportunities for etanercept treatment. Nephrol Dial Transplant. (2023) 38:1139–50. doi: 10.1093/ndt/gfac290, PMID: 36269313 PMC10157768

[91] Lindstrom NO Lawrence ML Burn SF Johansson JA Bakker ER Ridgway RA. Integrated beta-catenin, BMP, PTEN, and Notch signalling patterns the nephron. Elife. (2015) 4:e04000. doi: 10.7554/eLife.04000 PMC4337611 25647637

[92] Schunk SJ Floege J Fliser D Speer T. WNT-beta-catenin signalling – a versatile player in kidney injury and repair. Nat Rev Nephrol. (2021) 17:172–84. doi: 10.1038/s41581-020-00343-w, PMID: 32989282

[93] Lee H Fessler MB Qu P Heymann J Kopp JB. Macrophage polarization in innate immune responses contributing to pathogenesis of chronic kidney disease. BMC Nephrol. (2020) 21:270. doi: 10.1186/s12882-020-01921-7, PMID: 32660446 PMC7358194

[94] Cao Y Xiong J Guan X Yin S Chen J Yuan S. Paeoniflorin suppresses kidney inflammation by regulating macrophage polarization via KLF4-mediated mitophagy. Phytomedicine. (2023) 116:154901. doi: 10.1016/j.phymed.2023.154901, PMID: 37247587

[95] Wang X Xue N Zhao S Shi Y Ding X Fang Y. Upregulation of miR-382 contributes to renal fibrosis secondary to aristolochic acid-induced kidney injury via PTEN signaling pathway. Cell Death Dis. (2020) 11:620. doi: 10.1038/s41419-020-02876-1, PMID: 32796834 PMC7429500

[96] Sun D Cui S Ma H Zhu P Li N Zhang X. Salvianolate ameliorates renal tubular injury through the Keap1/Nrf2/ARE pathway in mouse kidney ischemia-reperfusion injury. J Ethnopharmacol. (2022) 293:115331. doi: 10.1016/j.jep.2022.115331 35489662

[97] Kashoor I Batlle D. Proximal renal tubular acidosis with and without Fanconi syndrome. Kidney Res Clin Pract. (2019) 38:267–81. doi: 10.23876/j.krcp.19.056, PMID: 31474092 PMC6727890

[98] Zhao S Li W Yu W Rao T Li H Ruan Y. Exosomal miR-21 from tubular cells contributes to renal fibrosis by activating fibroblasts via targeting PTEN in obstructed kidneys. Theranostics. (2021) 11:8660–73. doi: 10.7150/thno.62820, PMID: 34522205 PMC8419054

[99] Peng Y Liao K Tan F Liang Y Sun X Cui Z. Suppression of EZH2 inhibits TGF-beta1-induced EMT in human retinal pigment epithelial cells. Exp Eye Res. (2022) 222:109158. doi: 10.1016/j.exer.2022.109158, PMID: 35780904

[100] Zhu Q Dong H Bukhari AA Zhao A Li M Sun Y. HUWE1 promotes EGFR ubiquitination and degradation to protect against renal tubulointerstitial fibrosis. FASEB J. (2020) 34:4591–601. doi: 10.1096/fj.201902751R 32017279

[101] Choi D Kim CL Kim JE Mo JS Jeong HS. Hesperetin inhibit EMT in TGF-beta treated podocyte by regulation of mTOR pathway. Biochem Biophys Res Commun. (2020) 528:154–9. doi: 10.1016/j.bbrc.2020.05.087, PMID: 32451085

[102] Li Y Zhang Y Shi H Liu X Li Z Zhang J. CRTC2 activates the epithelial-mesenchymal transition of diabetic kidney disease through the CREB-Smad2/3 pathway. Mol Med. (2023) 29:146. doi: 10.1186/s10020-023-00744-0, PMID: 37884902 PMC10604535

[103] Allison SJ. Ubiquitylation of PTEN drives fibrosis in diabetic kidney disease. Nat Rev Nephrol. (2019) 15:254. doi: 10.1038/s41581-019-0130-y, PMID: 30804524

[104] Ding N Lv Y Su H Wang Z Kong X Zhen J. Vascular calcification in CKD: New insights into its mechanisms. J Cell Physiol. (2023) 238:1160–82. doi: 10.1002/jcp.31021, PMID: 37269534

[105] Hruska KA Mathew S Saab G. Bone morphogenetic proteins in vascular calcification. Circ Res. (2005) 97:105–14. doi: 10.1161/01.RES.00000175571.53833.6c 16037577

[106] Manzano-Lista FJ Sanz-Gomez M Gonzalez-Moreno D Vega-Martin E Gil-Ortega M Schulz A. Imbalance in Bone Morphogenic Proteins 2 and 7 Is Associated with Renal and Cardiovascular Damage in Chronic Kidney Disease. Int J Mol Sci. (2022) 24:40. doi: 10.3390/ijms24010040, PMID: 36613483 PMC9820638

[107] Ueda H Miyazaki Y Matsusaka T Utsunomiya Y Kawamura T Hosoya T. Bmp in podocytes is essential for normal glomerular capillary formation. J Am Soc Nephrol. (2008) 19:685–94. doi: 10.1681/ASN.2006090983, PMID: 18272846 PMC2390961

[108] Himmelfarb J Chertow GM McCullough PA Mesana T Shaw AD Sundt TM. Perioperative THR-184 and AKI after Cardiac Surgery. J Am Soc Nephrol. (2018) 29:670–9. doi: 10.1681/ASN.2017020217, PMID: 29203473 PMC5791058

[109] Srivastava A Singla DK. PTEN-AKT pathway attenuates apoptosis and adverse remodeling in ponatinib-induced skeletal muscle toxicity following BMP-7 treatment. Physiol Rep. (2023) 11:e15629. doi: 10.14814/phy2.15629, PMID: 36945866 PMC10031244

[110] Higgins DF Ewart LM Masterson E Tennant S Grebnev G Prunotto M. BMP7-induced-Pten inhibits Akt and prevents renal fibrosis. Biochim Biophys Acta Mol basis Dis. (2017) 1863:3095–104. doi: 10.1016/j.bbadis.2017.09.011, PMID: 28923783

[111] Baaten C Vondenhoff S Noels H. Endothelial Cell Dysfunction and Increased Cardiovascular Risk in Patients With Chronic Kidney Disease. Circ Res. (2023) 132:970–92. doi: 10.1161/CIRCRESAHA.123.321752, PMID: 37053275 PMC10097498

[112] Kajimoto H Kai H Aoki H Uchiwa H Aoki Y Yasuoka S. BMP type I receptor inhibition attenuates endothelial dysfunction in mice with chronic kidney disease. Kidney Int. (2015) 87:128–36. doi: 10.1038/ki.2014.223, PMID: 24963916

[113] Li J Yang L Qin W Zhang G Yuan J Wang F. Adaptive induction of growth differentiation factor 15 attenuates endothelial cell apoptosis in response to high glucose stimulus. PLoS One. (2013) 8:e65549. doi: 10.1371/journal.pone.0065549, PMID: 23799024 PMC3683015

[114] Sakshi R Saini A Verma C Mani I. Epigenetics in renal diseases. Prog Mol Biol Transl Sci. (2023) 198:61–71. doi: 10.1016/bs.pmbts.2023.02.013 37225324

[115] Liu Z Liu J Wang W An X Luo L Yu D. Epigenetic modification in diabetic kidney disease. Front Endocrinol. (2023) 14:1133970. doi: 10.3389/fendo.2023.1133970 PMC10348754 37455912

[116] Wang J Shen F Liu F Zhuang S. Histone Modifications in Acute Kidney Injury. Kidney Dis. (2022) 8:466–77. doi: 10.1159/000527799, PMID: 36590679 PMC9798838

[117] Li KY Tam CHT Liu H Day S Lim CKP So WY. DNA methylation markers for kidney function and progression of diabetic kidney disease. Nat Commun. (2023) 14:2543. doi: 10.1038/s41467-023-37837-7 37188670 PMC10185566

[118] Liu L Zou J Guan Y Zhang Y Zhang W Zhou X. Blocking the histone lysine 79 methyltransferase DOT1L alleviates renal fibrosis through inhibition of renal fibroblast activation and epithelial-mesenchymal transition. FASEB J. (2019) 33:11941–58. doi: 10.1096/fj.201801861R, PMID: 31373855 PMC8793787

[119] Zheng W Guo J Liu ZS. Effects of metabolic memory on inflammation and fibrosis associated with diabetic kidney disease: an epigenetic perspective. Clin Epigenetics. (2021) 13:87. doi: 10.1186/s13148-021-01079-5, PMID: 33883002 PMC8061201

[120] Cappuccilli M Bergamini C Giacomelli FA Cianciolo G Donati G Conte D. Vitamin B supplementation and nutritional intake of methyl donors in patients with chronic kidney sisease: a critical review of the impact on epigenetic machinery. Nutrients. (2020) 12:234. doi: 10.3390/nu12051234 32349312 PMC7281987

[121] Ike T Doi S Nakashima A Sasaki K Ishiuchi N Asano T. The hypoxia-inducible factor-alpha prolyl hydroxylase inhibitor FG4592 ameliorates renal fibrosis by inducing the H3K9 demethylase JMJD1A. Am J Physiol Renal Physiol. (2022) 323:F539–52. doi: 10.1152/ajprenal.00083.2022 36074918

[122] An C Jiao B Du H Tran M Song B Wang P. Jumonji domain-containing protein-3 (JMJD3) promotes myeloid fibroblast activation and macrophage polarization in kidney fibrosis. Br J Pharmacol. (2023) 180:2250–65. doi: 10.1111/bph.16096, PMID: 37076137 PMC12641593

[123] Yu C Xiong C Tang J Hou X Liu N Bayliss G. Histone demethylase JMJD3 protects against renal fibrosis by suppressing TGFbeta and Notch signaling and preserving PTEN expression. Theranostics. (2021) 11:2706–21. doi: 10.7150/thno.48679, PMID: 33456568 PMC7806480

[124] Fontecha-Barriuso M Martin-Sanchez D Ruiz-Andres O Poveda J Sanchez-Nino MD Valino-Rivas L. Targeting epigenetic DNA and histone modifications to treat kidney disease. Nephrol Dial Transplant. (2018) 33:1875–86. doi: 10.1093/ndt/gfy009 29534238

[125] Yoshimoto N Hayashi K Hishikawa A Hashiguchi A Nakamichi R Sugita-Nishimura E. Significance of podocyte DNA damage and glomerular DNA methylation in CKD patients with proteinuria. Hypertens Res. (2023) 46:1000–8. doi: 10.1038/s41440-023-01169-2, PMID: 36646881

[126] Hu T Chen F Chen D Liang H. DNMT3a negatively regulates PTEN to activate the PI3K/AKT pathway to aggravate renal fibrosis. Cell Signal. (2022) 96:110352. doi: 10.1016/j.cellsig.2022.110352, PMID: 35523401

[127] Samarakoon R Helo S Dobberfuhl AD Khakoo NS Falke L Overstreet JM. Loss of tumour suppressor PTEN expression in renal injury initiates SMAD3-and p53-dependent fibrotic responses. J Pathol. (2015) 236:421–32. doi: 10.1002/path.4538, PMID: 25810340 PMC4509814

[128] Guan H Zhu N Tang G Du Y Wang L Yuan W. DNA methyltransferase 1 knockdown reverses PTEN and VDR by mediating demethylation of promoter and protects against renal injuries in hepatitis B virus-associated glomerulonephritis. Cell Biosci. (2022) 12:98. doi: 10.1186/s13578-022-00835-1, PMID: 35765066 PMC9238139

[129] Zhang J Lee YR Dang F Gan W Menon AV Katon JM. PTEN Methylation by NSD2 Controls Cellular Sensitivity to DNA Damage. Cancer Discov. (2019) 9:1306–23. doi: 10.1158/2159-8290.CD-18-0083, PMID: 31217297 PMC6726527

[130] Zhang X Lan D Ning S Jia H Yu S. Botulinum toxin type A prevents the phenotypic transformation of fibroblasts induced by TGF-beta1 via the PTEN/PI3K/Akt signaling pathway. Int J Mol Med. (2019) 44:661–71. doi: 10.3892/ijmm.2019.4226, PMID: 31173164 PMC6605626

[131] Li Y Chen F Wei A Bi F Zhu X Yin S. Klotho recovery by genistein via promoter histone acetylation and DNA demethylation mitigates renal fibrosis in mice. J Mol Med. (2019) 97:541–52. doi: 10.1007/s00109-019-01759-z, PMID: 30806715

[132] Song Y Tao Q Yu L Li L Bai T Song X. Activation of autophagy contributes to the renoprotective effect of postconditioning on acute kidney injury and renal fibrosis. Biochem Biophys Res Commun. (2018) 504:641–6. doi: 10.1016/j.bbrc.2018.09.003, PMID: 30205956

[133] Gong W Luo C Peng F Xiao J Zeng Y Yin B. Brahma-related gene-1 promotes tubular senescence and renal fibrosis through Wnt/beta-catenin/autophagy axis. Clin Sci. (2021) 135:1873–95. doi: 10.1042/CS20210447, PMID: 34318888 PMC8358963

[134] Jin D Zhao Y Sun Y Xue J Li X Wang X. Jiedu Tongluo Baoshen formula enhances renal tubular epithelial cell autophagy to prevent renal fibrosis by activating SIRT1/LKB1/AMPK pathway. Biomed Pharmacother. (2023) 160:114340. doi: 10.1016/j.biopha.2023.114340 36738503

[135] Fu Y Xiang Y Li H Chen A Dong Z. Inflammation in kidney repair: Mechanism and therapeutic potential. Pharmacol Ther. (2022) 237:108240. doi: 10.1016/j.pharmthera.2022.108240 35803367

[136] Kramann R Kusaba T Humphreys BD. Who regenerates the kidney tubule? Nephrol Dial Transplant. (2015) 30:903–10. doi: 10.1093/ndt/gfu281, PMID: 25155054 PMC4438740

[137] Takahashi Y He H Tang Z Hattori T Liu Y Young MM. An autophagy assay reveals the ESCRT-III component CHMP2A as a regulator of phagophore closure. Nat Commun. (2018) 9:2855. doi: 10.1038/s41467-018-05254-w, PMID: 30030437 PMC6054611

[138] Shao R Wang X Xu T Xia Y Cui D. The balance between AIM2-associated inflammation and autophagy: the role of CHMP2A in brain injury after cardiac arrest. J Neuroinflammation. (2021) 18:257. doi: 10.1186/s12974-021-02307-8 34740380 PMC8571899

[139] Zhen Y Spangenberg H Munson MJ Brech A Schink KO Tan KW. ESCRT-mediated phagophore sealing during mitophagy. Autophagy. (2020) 16:826–41. doi: 10.1080/15548627.2019.1639301, PMID: 31366282 PMC7158923

[140] Wang H Wang Y Wang X Huang H Bao J Zhong W. PTEN alleviates maladaptive repair of renal tubular epithelial cells by restoring CHMP2A-mediated phagosome closure. Cell Death Dis. (2021) 12:1087. doi: 10.1038/s41419-021-04372-6, PMID: 34789720 PMC8599682

[141] Meng F Zhang Z Chen C Liu Y Yuan D Hei Z. PI3K/AKT activation attenuates acute kidney injury following liver transplantation by inducing FoxO3a nuclear export and deacetylation. Life Sci. (2021) 272:119119. doi: 10.1016/j.lfs.2021.119119, PMID: 33508296

[142] Di Meo A Batruch I Brown MD Yang C Finelli A Jewett MA. Searching for prognostic biomarkers for small renal masses in the urinary proteome. Int J Cancer. (2020) 146:2315–25. doi: 10.1002/ijc.32650, PMID: 31465112

[143] Wang J Shen T Zhu W Dou L Gu H Zhang L. Protein phosphatase 5 and the tumor suppressor p53 down-regulate each other's activities in mice. J Biol Chem. (2018) 293:18218–29. doi: 10.1074/jbc.RA118.004256, PMID: 30262665 PMC6254348

[144] Kma L Baruah TJ. The interplay of ROS and the PI3K/Akt pathway in autophagy regulation. Biotechnol Appl Biochem. (2022) 69:248–64. doi: 10.1002/bab.2104, PMID: 33442914

[145] Lee SK Lee JO Kim JH Kim SJ You GY Moon JW. Metformin sensitizes insulin signaling through AMPK-mediated PTEN down-regulation in preadipocyte 3T3-L1 cells. J Cell Biochem. (2011) 112:1259–67. doi: 10.1002/jcb.23000, PMID: 21465524

[146] Alqabandi M de Franceschi N Maity S Miguet N Bally M Roos WH. The ESCRT-III isoforms CHMP2A and CHMP2B display different effects on membranes upon polymerization. BMC Biol. (2021) 19:66. doi: 10.1186/s12915-021-00983-9, PMID: 33832485 PMC8033747

[147] Maidarti M Clarkson YL McLaughlin M Anderson RA Telfer EE. Inhibition of PTEN activates bovine non-growing follicles in vitro but increases DNA damage and reduces DNA repair response. Hum Reprod. (2019) 34:297–307. doi: 10.1093/humrep/dey354, PMID: 30521029 PMC6343469

[148] Chauhan A Sah DK Kumari N Kalra N Soni R Bhatt AN. PTEN inhibitor bpV(HOpic) confers protection against ionizing radiation. Sci Rep. (2021) 11:1720. doi: 10.1038/s41598-020-80754-8, PMID: 33462262 PMC7814022

[149] Al-Shahat A Hulail MAE Soliman NMM Khamis T Fericean LM Arisha AH. Melatonin Mitigates Cisplatin-Induced Ovarian Dysfunction via Altering Steroidogenesis, Inflammation, Apoptosis, Oxidative Stress, and PTEN/PI3K/Akt/mTOR/AMPK Signaling Pathway in Female Rats. Pharmaceutics. (2022) 14:769. doi: 10.3390/pharmaceutics14122769, PMID: 36559263 PMC9786155

[150] Terren C Nisolle M Munaut C. Pharmacological inhibition of the PI3K/PTEN/Akt and mTOR signalling pathways limits follicle activation induced by ovarian cryopreservation and in vitro culture. J Ovarian Res. (2021) 14:95. doi: 10.1186/s13048-021-00846-5 34275490 PMC8287691

[151] Dillon LM Miller TW. Therapeutic targeting of cancers with loss of PTEN function. Curr Drug Targets. (2014) 15:65–79. doi: 10.2174/1389450114666140106100909, PMID: 24387334 PMC4310752

[152] Moses C Nugent F Waryah CB Garcia-Bloj B Harvey AR Blancafort P. Activating PTEN Tumor Suppressor Expression with the CRISPR/dCas9 System. Mol Ther Nucleic Acids. (2019) 14:287–300. doi: 10.1016/j.omtn.2018.12.003, PMID: 30654190 PMC6348769

[153] Mahtal N Lenoir O Tinel C Anglicheau D Tharaux PL. MicroRNAs in kidney injury and disease. Nat Rev Nephrol. (2022) 18:643–62. doi: 10.1038/s41581-022-00608-6 35974169

[154] Belavgeni A Meyer C Stumpf J Hugo C Linkermann A. Ferroptosis and Necroptosis in the Kidney. Cell Chem Biol. (2020) 27:448–62. doi: 10.1016/j.chembiol.2020.03.016, PMID: 32302582

[155] Zhu Z Hu J Chen Z Feng J Yang X Liang W. Transition of acute kidney injury to chronic kidney disease: role of metabolic reprogramming. Metabolism. (2022) 131:155194. doi: 10.1016/j.metabol.2022.155194, PMID: 35346693

[156] Wang G Lunardi A Zhang J Chen Z Ala U Webster KA. Zbtb7a suppresses prostate cancer through repression of a Sox9-dependent pathway for cellular senescence bypass and tumor invasion. Nat Genet. (2013) 45:739–46. doi: 10.1038/ng.2654, PMID: 23727861 PMC4036521

[157] Wang X Xie Z Lou Z Chen Y Huang S Ren Y. Regulation of the PTEN/PI3K/AKT pathway in RCC using the active compounds of natural products in vitro. Mol Med Rep. (2021) 24:406. doi: 10.3892/mmr.2021.12406 PMC8430319 34490473

[158] Xu H Kong Y Chen Y Li N Zhang S Lu H. Natural Plant Extract Berbamine Is a Potent Inhibitor of Cell Growth and Survival of Human Tenon's Fibroblasts. Ophthalmic Res. (2020) 63:555–63. doi: 10.1159/000506644 32079013

[159] Wang Z Liu F Liao W Yu L Hu Z Li M. Curcumin suppresses glioblastoma cell proliferation by p-AKT/mTOR pathway and increases the PTEN expression. Arch Biochem Biophys. (2020) 689:108412. doi: 10.1016/j.abb.2020.108412, PMID: 32445778

[160] Ashrafizadeh M Zarrabi A Hashemipour M Vosough M Najafi M Shahinozzaman M. Sensing the scent of death: Modulation of microRNAs by Curcumin in gastrointestinal cancers. Pharmacol Res. (2020) 160:105199. doi: 10.1016/j.phrs.2020.105199, PMID: 32942019

[161] Mirzaei H Masoudifar A Sahebkar A Zare N Sadri Nahand J Rashidi B. MicroRNA: A novel target of curcumin in cancer therapy. J Cell Physiol. (2018) 233:3004–15. doi: 10.1002/jcp.26055, PMID: 28617957

[162] Liang D Wen Z Han W Li W Pan L Zhang R. Curcumin protects against inflammation and lung injury in rats with acute pulmonary embolism with the involvement of microRNA-21/PTEN/NF-kappaB axis. Mol Cell Biochem. (2021) 476:2823–35. doi: 10.1007/s11010-021-04127-z, PMID: 33730297

